# Plasma sTNFR1 and IL8 for prognostic enrichment in sepsis trials: a prospective cohort study

**DOI:** 10.1186/s13054-019-2684-2

**Published:** 2019-12-09

**Authors:** Brian J. Anderson, Carolyn S. Calfee, Kathleen D. Liu, John P. Reilly, Kirsten N. Kangelaris, Michael G. S. Shashaty, Aili L. Lazaar, Andrew I. Bayliffe, Robert J. Gallop, Todd A. Miano, Thomas G. Dunn, Erik Johansson, Jason Abbott, Alejandra Jauregui, Thomas Deiss, Kathryn Vessel, Annika Belzer, Hanjing Zhuo, Michael A. Matthay, Nuala J. Meyer, Jason D. Christie

**Affiliations:** 1 Division of Pulmonary, Allergy and Critical Care Medicine, Perelman School of Medicine, University of Pennsylvania, 3400 Spruce Street, 5036 Gates Building, Philadelphia, PA 19104 USA; 2 Division of Pulmonary and Critical Care Medicine, University of California San Francisco, San Francisco, USA; 3 Division of Hospital Medicine, Department of Medicine, University of California San Francisco, San Francisco, USA; 4 Center for Clinical Epidemiology and Biostatistics, Perelman School of Medicine, University of Pennsylvania, Philadelphia, USA; 5 Department of Medicine, Perelman School of Medicine, University of Pennsylvania, Philadelphia, USA; 6 GlaxoSmithKline R&D, Brentford, UK; 7 Department of Mathematics, West Chester University, West Chester, USA

**Keywords:** Sepsis, Tumor necrosis factor receptors, Interleukin-8, Angiopoietin-2, Biomarkers, Prognostic enrichment

## Abstract

**Background:**

Enrichment strategies improve therapeutic targeting and trial efficiency, but enrichment factors for sepsis trials are lacking. We determined whether concentrations of soluble tumor necrosis factor receptor-1 (sTNFR1), interleukin-8 (IL8), and angiopoietin-2 (Ang2) could identify sepsis patients at higher mortality risk and serve as prognostic enrichment factors.

**Methods:**

In a multicenter prospective cohort study of 400 critically ill septic patients, we derived and validated thresholds for each marker and expressed prognostic enrichment using risk differences (RD) of 30-day mortality as predictive values. We then used decision curve analysis to simulate the prognostic enrichment of each marker and compare different prognostic enrichment strategies.

**Measurements and main results:**

An admission sTNFR1 concentration > 8861 pg/ml identified patients with increased mortality in both the derivation (RD 21.6%) and validation (RD 17.8%) populations. Among immunocompetent patients, an IL8 concentration > 94 pg/ml identified patients with increased mortality in both the derivation (RD 17.7%) and validation (RD 27.0%) populations. An Ang2 level > 9761 pg/ml identified patients at 21.3% and 12.3% increased risk of mortality in the derivation and validation populations, respectively. Using sTNFR1 or IL8 to select high-risk patients improved clinical trial power and efficiency compared to selecting patients with septic shock. Ang2 did not outperform septic shock as an enrichment factor.

**Conclusions:**

Thresholds for sTNFR1 and IL8 consistently identified sepsis patients with higher mortality risk and may have utility for prognostic enrichment in sepsis trials.

## Introduction

Sepsis carries a high mortality and has limited pharmacologic therapy [1]. A pipeline of therapies targeting inflammation, vascular regulation, and immune regulation are in development, often in tandem with the oncology, autoimmune, and cardiovascular spheres [2, 3]. However, these therapies may be associated with adverse effects that tip the risk-benefit scale in favor of testing the new therapy among high-risk patients first, to avoid exposing low-risk patients to a potentially risky therapy. In addition, prior sepsis trials have been hampered by imprecise estimates of baseline mortality [4], making interpretation challenging due to inadequate power.

Several fields have embraced enrichment strategies to refine patient selection for clinical trials, fostering the translation of experimental therapies [5–8]. This includes “prognostic enrichment,” selecting patients with a greater likelihood of having an outcome, and “predictive enrichment,” selecting patients with a greater likelihood of responding to a specific intervention [9–11]. Despite broad appeal, enrichment strategies have not been widely applied in critical care, partly due to a dearth of biomarkers that might serve as enrichment factors [12–15].

We sought to develop a simple biomarker-based prognostic enrichment method for selecting high-risk patients for future sepsis trials. We chose three markers, soluble tumor necrosis factor receptor-1 (sTNFR1), interleukin-8 (IL8), and angiopoietin-2 (Ang2), which are easily measurable, associate with sepsis outcomes, and represent pathways that are potential targets for sepsis therapy [16–22].

To assess each marker’s prognostic enrichment potential, we derived concentration thresholds and validated whether the thresholds consistently identified subjects at higher mortality risk in two separate cohorts using risk differences as predictive values. Additionally, we used decision curve analysis to illustrate the potential benefits of using each marker to select subjects for clinical trials.

## Methods

### Study design

Detailed methods are provided in the supplement (Additional file 1). We performed a multicenter prospective cohort study enrolling critically ill septic patients admitted from the emergency departments at the University of Pennsylvania (PENN) and the University of California San Francisco (UCSF). Both cohorts have been described previously [23, 24]. We chose a priori to enroll 200 subjects at each site during the same period. The primary outcome was 30-day mortality. We defined immunocompromise using Acute Physiology, Age, Chronic Health Evaluation (APACHE) criteria and acute respiratory distress syndrome (ARDS) using Berlin criteria [25–27]. We defined septic shock as the receipt of vasopressors and a lactate > 2 mmol/l on the day of intensive care unit (ICU) admission [1]. Each cohort was approved by its institution’s Institutional Review Board.

### Plasma protein measurement

Plasma was obtained as close to ICU bed request as feasible and within 24 h of ICU admission. We measured sTNFR1, IL8, and Ang2 concentrations using enzyme-linked immunosorbent assays (R&D Systems).

### Statistical methods

We first confirmed each marker’s prognostic value by determining whether the marker was independently associated with mortality and whether the marker improved model fit (likelihood-ratio test) and discrimination (area under the receiver operating characteristic curve [AUC]) when added to a clinical variable model for mortality. We chose variables that were easily available at admission and associated with mortality, including age, diabetes, cirrhosis, immunocompromise, septic shock, and mechanical ventilation. To operationalize each marker, we split the population by enrollment site and derived thresholds for each marker in the derivation population (PENN) using the Youden method [28]. We performed logistic regression adjusting for the variables above and calculated standardized risks and risk differences (RD) for mortality between marker-positive and marker-negative subjects [29]. For validation, we simulated the effect of simply applying each marker threshold in the validation population (UCSF) without clinical variables and focused on whether the unadjusted RD fell within the 95% confidence interval (CI) of the standardized RD in the derivation population, indicating the marker identified high-risk patients in a similar fashion while accounting for differences in baseline mortality [30]. We secondarily performed adjusted analyses in the validation population and tested whether each marker threshold improved model fit and discrimination when added to a clinical variable model for mortality in each population. We tested for effect modification by immunocompromised status because differences in inflammatory pathways have been reported in these patients [24].

Next, we employed decision curve analysis (DCA) to illustrate each marker’s potential as an enrichment factor (i.e., enroll if marker-positive) [31–33]. The net benefit (percent true positives − percent false positives) was calculated across a range of threshold probabilities for mortality, where the threshold probability represents the mortality risk a trial would set for enrollment (i.e., enroll patients with ≥ 35% mortality risk). Decision curves are interpreted vertically; at each threshold probability, the strategy with the highest net benefit identifies the highest number of true positives relative to false positives and thus selects the most efficient trial population. We compared five enrichment strategies: (1) enrolling all sepsis patients (no enrichment), (2) enrolling septic shock patients, (3) enrolling patients positive for a single marker, (4) enrolling patients positive for two markers, and (5) enrolling patients whose predicted mortality using the markers as continuous variables met a certain threshold [22]. To further illustrate enrichment potential, we calculated sample sizes for a hypothetical trial testing a therapy with a 20% relative risk reduction of mortality, assuming 90% power. We chose septic shock as our primary clinical variable enrichment factor because it is often used to define a high-risk subgroup in sepsis trials [34, 35]. Secondarily, we evaluated an APACHE II score ≥ 20 and a peak lactate ≥ 4 mmol/l within the first 24 h as clinical variable prognostic enrichment factors [26, 36].

Analyses were performed using Stata 15.1; a two-sided *p* < 0.05 was considered statistically significant.

## Results

### Patient characteristics

We enrolled 400 critically ill septic patients (Additional file 2: Figure S1); baseline characteristics are summarized in Table 1. The derivation population was younger, more immunocompromised, had less septic shock, more African-American subjects, and fewer Asian subjects. Both populations had significant 30-day mortality, 41.0% and 27.0% in the derivation and validation populations, respectively. Plasma concentrations of sTNFR1 and IL8 were higher in the derivation population.

**Table 1:** Characteristics of study population in the derivation and validation cohorts

Variable	Derivation (*n* = 200)	Validation (*n* = 200)	*p*
Age	60 (49.5–69)	67 (59–78)	< 0.001
Male gender	110 (55.0%)	105 (52.5%)	0.62
Race			
Caucasian	110 (55.0%)	106 (53%)	< 0.001
African American	82 (41.0%)	24 (12.0%)	
Asian	4 (2.0%)	50 (25.0%)	
Other	4 (2.0%)	20 (10.0%)	
Diabetes mellitus	59 (29.5%)	57 (28.5%)	0.83
Cirrhosis	20 (10.0%)	17 (8.5%)	0.61
Immunocompromised	95 (47.5%)	27 (13.5%)	< 0.001
Pneumonia	78 (39.0%)	104 (52.5%)	0.007
APACHE II	25 (19.5–32.5)	25 (19–33)	0.99
Septic shock at presentation	77 (38.5%)	98 (49.0%)	0.034
Invasive ventilation at presentation	82 (41.0%)	82 (41.0%)	1.0
ARDS	57 (28.5%)	50 (25.3%)	0.47
30-day mortality	82 (41.0%)	54 (27.0%)	0.003
sTNFR1 (pg/ml)	8444 (4332–13,450)	6366 (3232–11,024)	0.004
IL8 (pg/ml)	115.7 (51.2–325.6)	54.7 (23.5–241.0)	< 0.001
Ang2 (pg/ml)	13,933 (8747–26,865)	13,894 (7146–24,447)	0.24

*Abbreviations*: *APACHE* Acute Physiology, Age and Chronic Health Evaluation, *ARDS* acute respiratory distress syndrome, *sTNFR1* soluble tumor necrosis factor receptor-1, *IL* interleukin, *Ang2* angiopoietin-2

### Soluble tumor necrosis factor receptor-1

The plasma sTNFR1 concentration at ICU admission independently associated with mortality (OR [95% CI] per 1-log increase 1.68 [1.23–2.28]; *p* = 0.001), and adding the sTNFR1 concentration to a clinical variable model for mortality improved model fit and marginally improved discrimination (Additional file 2: Table S1). The optimal sTNFR1 threshold in the derivation population was 8861 pg/ml; 46.5% of patients were sTNFR1-positive with a 21.6% (95% CI 8.1–35.2; *p* = 0.002) adjusted increased absolute risk of mortality. In the validation population, 33.5% were sTNFR1-positive with a 17.8% (95% CI 4.2–31.3; *p* = 0.010) unadjusted increased absolute risk of mortality, which was within the 95% CI of the RD in the derivation population (Table 2). In adjusted analyses, the RD in the validation population was 13.0% (95% CI 0.3–25.7; *p* = 0.045). The sTNFR1 threshold improved model fit and marginally improved discrimination when added to a clinical variable model for mortality in each population (Additional file 2: Table S2).

**Table 2:** Risks and risk differences of 30-day mortality categorized by marker positivity for soluble tumor necrosis factor receptor-1 (sTNFR1), interleukin-8 (IL8), and angiopoietin-2 (Ang2), in the derivation (*N* = 200) and validation (*N* = 200) cohorts. Standardized risks and risk differences are reported for the derivation cohort, adjusted for age, cirrhosis, immunocompromised state, septic shock at presentation, and mechanical ventilation at presentation. Crude risks and risk differences are reported for the validation cohort. The IL8 analysis is limited to immunocompetent patients (*N* = 105 in derivation cohort, *N* = 173 in validation cohort)

Marker and site	Number (%) of subjects above threshold	30-day mortality (95% CI) if below threshold	30-day mortality (95% CI) if above threshold	Risk difference of 30-day mortality (95% CI) if above threshold	*p*
sTNFR1 > 8861 pg/ml					
Derivation	93 (46.5%)	30.4% (21.6, 39.2)	52.0% (42.3, 61.7)	21.6% (8.1, 35.2)	0.002
Validation	67 (33.5%)	21.1% (14.1, 28.0)	38.8% (27.1, 50.5)	17.8% (4.2, 31.3)	0.010
IL8 > 94 pg/ml					
Derivation	57 (54.3%)	23.2% (11.8, 34.6)	40.9% (29.8, 52.0)	17.7% (1.6, 33.8)	0.031
Validation	68 (39.3%)	18.9% (11.9, 25.8)	44.1% (32.3, 55.9)	27.0% (13.2, 40.8)	< 0.001
Ang2 > 9761 pg/ml					
Derivation	139 (69.5%)	25.8% (14.6, 37.1)	47.1% (39.3, 54.9)	21.3% (7.3, 35.3)	0.003
Validation	127 (63.5%)	19.2% (10.2, 28.2)	31.5% (23.4, 39.6)	12.3% (0.2, 24.4)	0.046

**Fig. 1:**
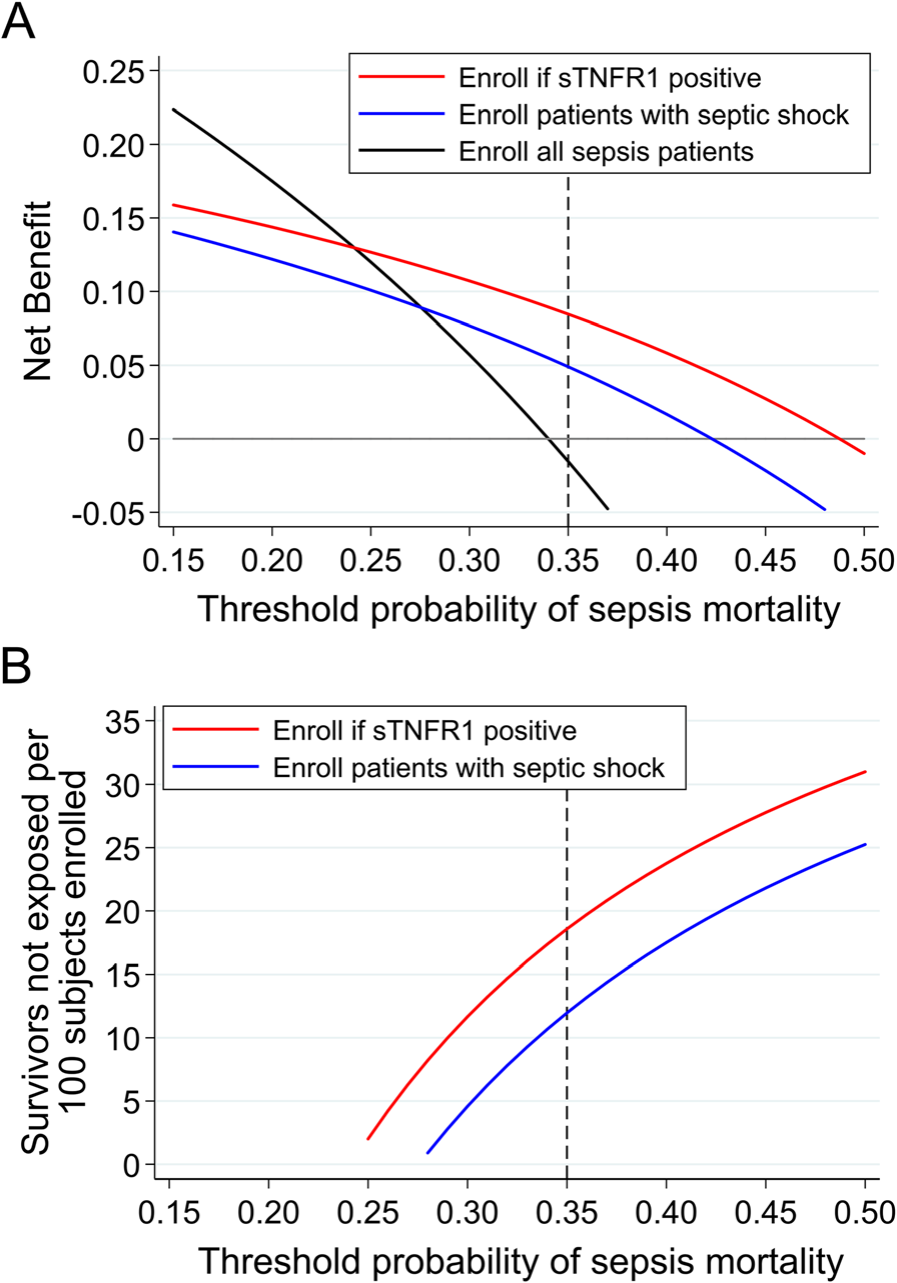
**a** Net benefit curves of three clinical trial enrollment strategies: enrolling all sepsis patients (black line), enrolling patients with septic shock (blue line), and enrolling sTNFR1 positive patients (red line). The *x*-axis represents the threshold probability, which is the probability of mortality that a hypothetical trial would require for enrollment. The *y*-axis is the net benefit, which represents the tradeoff between true positives and false positives, and is calculated as (true positives/*n*) − (false positive/*n*) × (*p*_t_/1 − *p*_t_), where *p*_t_ is the threshold probability. The net benefit varies with the threshold probability since it reflects the relative harms of missing non-survivors (false negatives) and enrolling too many survivors (false positives). We focused on threshold probabilities between 15 and 50%, because enrichment is unnecessary at low thresholds given baseline mortality, and we reasoned that patients with > 50% mortality risk may be excluded from trials because they may be less likely to respond to therapy. Net benefit curves are interpreted vertically; at each threshold probability, the strategy with the highest net benefit is the optimal strategy for enriching a trial with high-risk subjects. For example, if a trial sought to enroll patients with at least 35% mortality risk (dotted vertical line), enrolling only sTNFR1-positive patients is the optimal strategy. **b** Intervention curves comparing enrolling all sepsis patients (reference, not shown), enrolling patients with septic shock (blue line), and enrolling sTNFR1 positive patients (red line). Intervention curves are an alternative representation of net benefit and are also interpreted vertically. The *y*-axis represents the number of survivors that avoid the intervention, which in this case is enrollment and exposure to unproven therapy. At a threshold probability of 35%, enrolling sTNFR1-positive patients would lead to a greater reduction in the number of survivors unnecessarily exposed compared to enrolling patients with septic shock

For prognostic enrichment, enrolling sTNFR1-positive patients was superior to enrolling patients with septic shock based on test characteristics (positive predictive value [PPV] 48.8% vs. 42.3%; negative predictive value [NPV] 75.8% vs. 72.4%; Additional file 2: Table S3) and DCA. As shown in Fig. 1a, if a trial sought to enroll patients with < 25% mortality risk, enrolling all sepsis patients was optimal and no enrichment was needed. However, if a trial sought patients at higher mortality risk, i.e., ≥ 35%, enrolling sTNFR1-positive patients was superior to enrolling septic shock patients or enrolling all sepsis patients. In terms of efficiency, if a trial sought to enroll patients with ≥ 35% mortality risk, enrolling sTNFR1-positive patients would result in a strategy equivalent to 18 fewer survivors exposed per 100 patients enrolled, whereas enrolling septic shock patients would result in 12 fewer survivors exposed, compared to enrolling all sepsis patients (Fig. 1b, Additional file 2: Table S5). In terms of statistical power for a trial testing a therapy with a 20% relative risk reduction of mortality, enrolling sTNFR1-positive patients would reduce the required sample size by 43.3% (*N* = 1126), whereas enrolling septic shock patients would reduce it by 28.1% (*N* = 1428), compared to enrolling all sepsis patients (*N* = 1986).

### Interluekin-8

The plasma IL8 concentration at ICU admission independently associated with mortality (OR [95% CI] per 1-log increase 1.25 [1.09–1.43]; *p* = 0.001) and improved model fit and marginally improved discrimination when added to a clinical variable model for mortality (Additional file 2: Table S1). The optimal IL8 threshold was 94 pg/ml. We found effect modification by immunocompromised status on the IL8-mortality association (*p* = 0.033), with the association driven by immunocompetent patients (Additional file 2: Table S6). In the derivation population, 54.3% of immunocompetent patients were IL8-positive with a 17.7% (95% CI 1.6–33.8; *p* = 0.031) adjusted increased absolute risk of mortality. In the validation population, 39.3% of immunocompetent subjects were IL8-positive with a 27.0% (95% CI 13.2–40.8; *p* < 0.001) unadjusted absolute increased risk of mortality, which was within the 95% CI of the RD in the derivation population (Table 2). When adjusted for clinical variables, the RD in the validation population was 22.1% (95% CI 7.4–36.8; *p* = 0.003). The IL8 threshold improved model fit and marginally improved discrimination when added to a clinical variable model for mortality in each population (Additional file 2: Table S2).

**Fig. 2:**
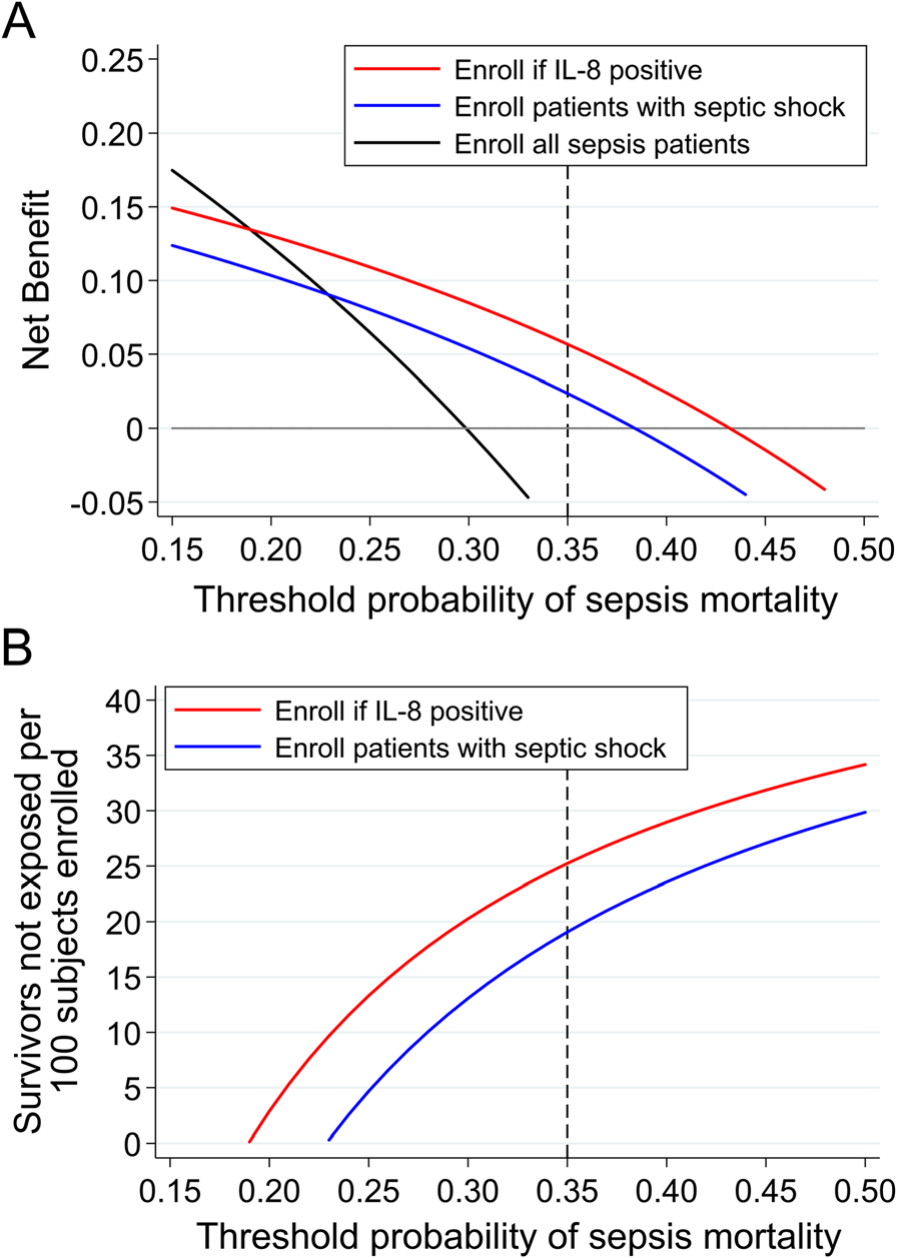
**a** Net benefit curves of three clinical trial enrollment strategies, enrolling all sepsis patients (black line), enrolling patients with septic shock (blue line), and enrolling IL-8-positive patients (red line), in a population restricted to immunocompetent patients. The *x*-axis represents the threshold probability, which is the probability of sepsis mortality that a hypothetical trial would require for enrollment. The *y*-axis is the net benefit, which represents the tradeoff between true positives and false positives, and is described further in the legend of Fig. 1. The net benefit curves are interpreted vertically, such that at each threshold probability, the strategy with the highest net benefit is the optimal strategy for enriching a trial with high-risk subjects. For example, if a trial sought to enroll patients with at least 35% mortality risk (dotted vertical line), enrolling only IL-8 positive patients is the optimal strategy. **b** Intervention curves comparing enrolling all sepsis patients (reference, not shown), enrolling patients with septic shock (blue line), and enrolling IL-8-positive patients (red line), in a population restricted to immunocompetent patients. The *y*-axis represents the number of survivors that avoid the intervention, which in this case is enrollment and exposure to unproven therapy. Intervention curves are also interpreted vertically. For example, at a threshold probability of 35%, enrolling only IL-8-positive patients would lead to a greater reduction in the number of survivors unnecessarily exposed compared to enrolling patients with septic shock

Among immunocompetent patients, the IL8 threshold was the superior prognostic enrichment factor compared to septic shock based on test characteristics (PPV 43.2% vs. 38.4%; NPV 81.7% vs. 77.1%; Additional file 2: Table S4) and DCA. As shown in Fig. 2a, if a trial sought to enroll patients with < 20% mortality risk, enrolling all sepsis patients was optimal; however, if a trial sought patients at higher mortality risk, i.e., ≥ 35%, enrolling IL8-positive patients was optimal. For example, if a trial sought to enroll patients with ≥ 35% mortality risk, enrolling IL8-positive patients would result in a strategy equivalent to 25 fewer survivors exposed per 100 patients enrolled, whereas enrolling septic shock patients would result in 19 fewer survivors exposed, compared to enrolling all sepsis patients (Fig. 2b, Additional file 2: Table S7). In terms of statistical power, enrolling IL8-positive patients would reduce the required sample size by 41.8% (*N* = 1380), whereas enrolling septic shock patients would reduce it by 30.0% (*N* = 1660), compared to enrolling all sepsis patients (*N* = 2372).

### Angiopoietin-2

**Fig. 3:**
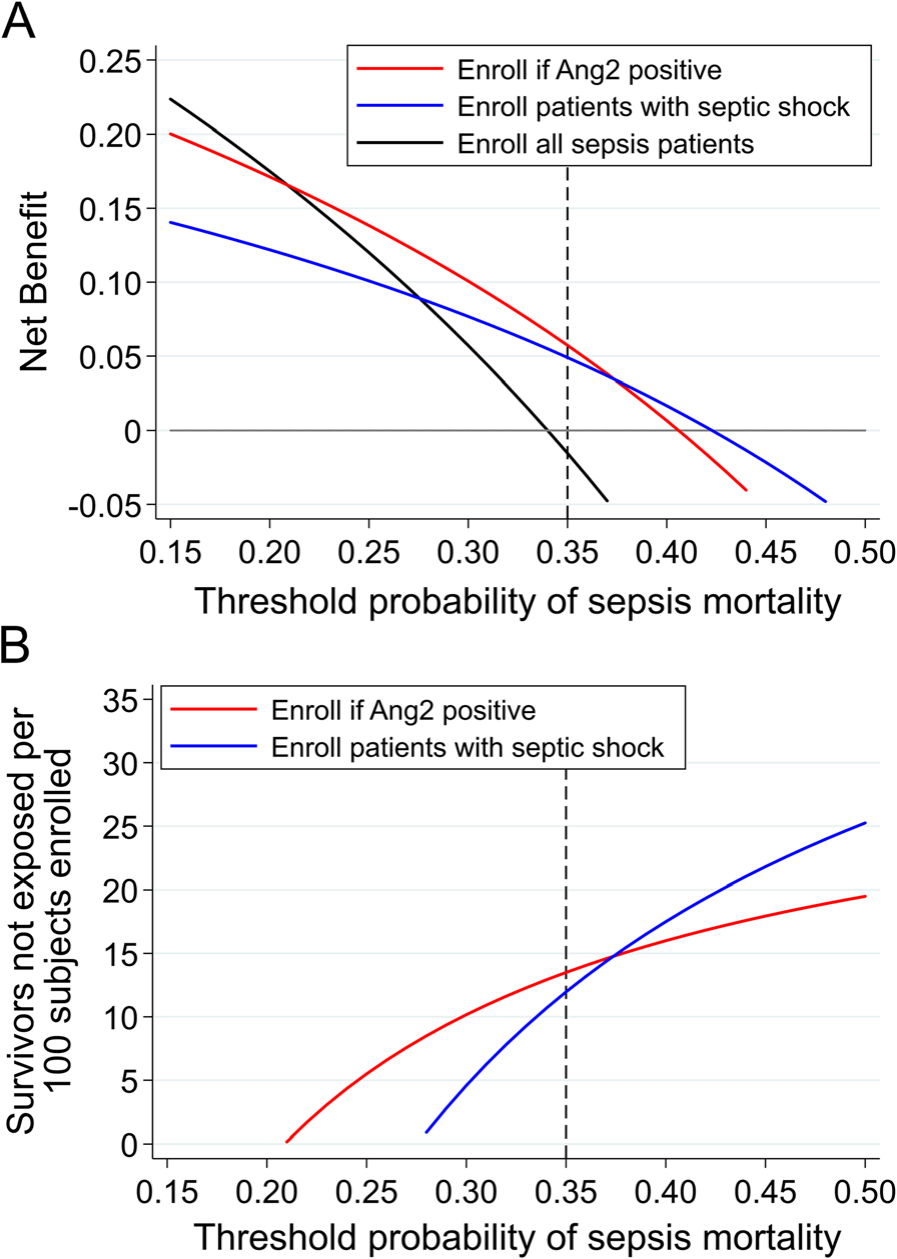
**a** Net benefit curves of three clinical trial enrollment strategies: enrolling all sepsis patients (black line), enrolling patients with septic shock (blue line), and enrolling Ang-2 positive patients (red line). The *x*-axis represents the threshold probability, which is the probability of sepsis mortality that a hypothetical trial would require for enrollment. The *y*-axis is the net benefit, which represents the tradeoff between true positives and false positives, and is described further in the legend of Fig. 1. The net benefit curves are interpreted vertically, such that at each threshold probability, the strategy with the highest net benefit is the optimal strategy for enriching a trial with high-risk subjects. For example, if a trial sought to enroll patients with at least 35% mortality risk (dotted vertical line), enrolling only Ang-2 positive patients had a similar net benefit to enrolling only patients with septic shock. **b** Intervention curves comparing enrolling all sepsis patients (reference, not shown), enrolling patients with septic shock (blue line), and enrolling Ang-2 positive patients (red line). Intervention curves are an alternative representation of net benefit. The *y*-axis represents the number of survivors that avoid the intervention, which in this case is enrollment and exposure to unproven and potentially risky therapy. Intervention curves are also interpreted vertically. For example, at a threshold probability of 35%, enrolling only Ang-2-positive patients led to a similar reduction in the number of survivors exposed when compared to enrolling only patients with septic shock

The plasma Ang2 concentration at ICU admission independently associated with mortality (OR [95% CI] per 1-log increase 1.53 [1.16–2.01]; *p* = 0.002) and improved model fit and marginally improved discrimination when added to a clinical variable model for mortality (Additional file 2: Table S1). The optimal Ang2 threshold was 9761 pg/ml. In the derivation population, 69.5% were Ang2-positive with a 21.3% (95% CI 7.3–35.3; *p* = 0.003) adjusted increased absolute risk of mortality. In the validation population, 63.5% were Ang2-positive with a 12.3% (95% CI 0.2–24.4; *p* = 0.046) unadjusted increased absolute risk of mortality (Table 2). In adjusted analyses, the RD in the validation population was 10.8% (95% CI − 1.1–22.6; *p* = 0.075). The Ang2 threshold did not consistently improve model fit and discrimination (Additional file 2: Table S2) and was not consistently superior to septic shock for prognostic enrichment (Fig. 3, Additional file 2: Tables S3 and S8).

### Combinatorial and secondary models

Secondarily, we evaluated the combined enrichment potential of sTNFR1 and IL8. Positivity for both sTNFR1 and IL8 improved discrimination to a similar degree as the individual markers (Additional file 2: Table S9), and as an enrichment factor performed similarly to sTNFR1 alone and slightly outperformed IL8 alone (Additional file 2: Figure S2). This may be due to the effect modification of immunocompromised status on the IL8-mortality association, as positivity for both sTNFR1 and IL8 was superior when restricted to immunocompetent patients (Additional file 2: Figure S3). Using the predicted mortality from sTNFR1 and IL8 concentrations as continuous variables yielded similar discrimination as the individual marker thresholds, similar enrichment as positivity for sTNFR1 alone, and slightly superior enrichment as positivity for IL8 alone (Additional file 2: Table S9, Figure S4).

For our secondary clinical variable enrichment methods, an APACHE II score ≥ 20 yielded superior enrichment compared to sTNFR1 positivity and IL8 positivity at lower mortality thresholds, whereas sTNFR1 positivity was superior to an APACHE II ≥ 20 at mortality thresholds above 33% and IL8 positivity was superior at mortality thresholds above 36% (Additional file 2: Table S3, Figure S5). Positivity for sTNFR1 outperformed peak lactate ≥ 4 mmol/l, whereas IL8 positivity performed similarly (Additional file 2: Table S3, Figure S6).

## Discussion

We found that plasma sTNFR1 and IL8 thresholds consistently identified subjects at higher mortality risk in two distinct populations of critically ill septic patients. We also demonstrated that sTNFR1 and IL8 could potentially serve as prognostic enrichment factors for sepsis trials. By selecting high-risk patients, using these markers could improve trial efficiency and power and reduce the number of survivors unnecessarily exposed to potentially risky therapies even more so than using septic shock.

Our results are consistent with a study by Mikacenic et al., which found that using sTNFR1 and IL8 concentrations in a continuous fashion could risk stratify sepsis patients [22]. Our data build on these findings by demonstrating that thresholds of these markers could provide a simpler method to refine trial enrollment, that the individual markers have enrichment potential and perform similarly to combined models, that the markers are superior to using septic shock for enrichment, and that the markers have prognostic value in patients with higher illness severity and mortality. Our data also reveal potential limitations of using IL8 for enrichment in immunocompromised patients, given our findings that the IL8 threshold was associated with higher mortality in immunocompetent subjects but not immunocompromised subjects.

Although we demonstrated that the Ang2 threshold identified high-risk patients, it did not appear to have utility over using septic shock for prognostic enrichment. This may be due to the association of dysregulated Ang2 with septic shock [37], suggesting both variables identified a similar high-risk subgroup and thus provided similar enrichment.

Our data add to the growing body of literature highlighting the potential benefits of biomarker-based enrichment for critical care trials. Recent sepsis trials have demonstrated a discrepancy between the estimated mortality based on clinical criteria and observed mortality [4, 38], which could result in inadequate power to detect a benefit of a tested therapy. Despite differing baseline mortality at our two sites, sTNFR1 positivity consistently identified subjects with at least 17.8% higher mortality risk, and IL8 positivity consistently identified immunocompetent patients with at least 17.7% higher mortality risk. These markers could potentially provide a method to ensure adequate baseline mortality in future trials.

Our study has several strengths. We performed a two-center cohort study, enrolling two distinct populations with a wide distribution of ages, diverse racial makeup, and differences in illness severity. Our use of plasma thresholds simulated operationalizing the markers as simple methods for trial enrichment. The biomarkers were measured as close to admission as possible, demonstrating they have prognostic relevance in early sepsis when most trials seek to enroll patients. We used risk differences to provide easily interpretable predictive values and employed decision curves, a novel method to evaluate biomarkers [39, 40], to compare enrichment strategies.

Our study also has limitations. Although we successfully derived and validated thresholds of each marker that consistently identified patients at higher risk of mortality in both cohorts, the improvements in discrimination when the markers were added to clinical variables were relatively incremental. In addition, although the marker thresholds outperformed an APACHE-based method at higher mortality thresholds, the APACHE-based method was superior at lower mortality thresholds. This may ultimately limit each marker’s utility as simple prognostic enrichment factors, given the APACHE score can be obtained from clinical data at no additional cost, and several barriers still need to be addressed to improve the feasibility and practicality of employing biomarker enrichment strategies, such as advances in rapid testing and validation of testing for clinical use [15]. In addition, we identified that the IL8 threshold’s utility as a prognostic enrichment factor was limited to immunocompetent patients, further limiting its potential as a sole enrichment factor given the frequency of sepsis among immunocompromised patients and because immune impairment may be unrecognized at the time of ICU admission. Although we selected these markers because of their prior association with sepsis mortality and their representation of pathways for which therapies are being developed, using these markers for prognostic enrichment may inadvertently select mechanistically distinct subgroups, which could limit the generalizability of clinical trials that use these markers for prognostic enrichment. Furthermore, other plasma markers and clinical variable enrichment methods may deserve consideration as prognostic enrichment factors in future studies.

We also recognize that the confidence intervals for the risk differences in mortality were moderately wide. However, the goal of using risk differences as predictive values was not for individual prognostication, but only to guide trial enrollment. Given the strength of the association in two distinct cohorts and the decision curve analysis demonstrating the potential value of using each marker when compared to clinical variable methods, these markers appear to have potential as prognostic enrichment factors. Larger studies may be needed to confirm our findings and refine the use of these markers as prognostic enrichment factors, which may include further refining the biomarker thresholds. It is also important to note that the mortality among marker-negative patients (false negatives) was not inconsequential. However, one benefit of DCA is that it does not make assumptions about the relative harms of false positives and false negatives, leaving it to the trialist to define their importance by choosing the threshold probability. If a trialist’s priority was minimizing the exclusion of non-survivors (i.e., minimizing false negatives), as would be the case for a trial of an inexpensive, low-risk therapy with a high likelihood of benefit, the trialist would set a low mortality threshold (i.e., enroll all patients with sepsis). Alternatively, if a trialist was concerned about potentially high-risk side effects from an experimental therapy, they might consider it necessary to exclude patients with low mortality risk and set a higher threshold even though some non-survivors would be excluded. Thus, the relative importance of false negatives and false positives varies based on the probability threshold, and the DCA provides a method for comparing enrollment strategies at each threshold.

Lastly, an important limitation of our study is that we were unable to evaluate the predictive enrichment potential of sTNFR1, IL8, and Ang2. Sepsis is a heterogeneous syndrome with myriad pathways contributing to organ dysfunction and death. Several recent studies have highlighted heterogeneity in the treatment effect of therapies in sepsis and suggest biomarkers could identify patients more likely to respond to therapy. In a prospective trial, sepsis patients with higher baseline interleukin-6 levels appeared more likely to respond to anti-tumor necrosis factor therapy [41]. In retrospective analyses, biomarker-based strategies identified heterogeneity in treatment effect in sepsis trials of recombinant human interleukin-1 receptor antagonist and anti-TNF-α antibody therapy [42, 43]. Similarly, studies have shown heterogeneity in treatment effect among patients with a hyperinflammatory subphenotype of ARDS [44–46]. Because sTNFR1, IL8, and Ang2 reflect pathways that are dysregulated in sepsis and for which therapies are being investigated, such as monoclonal antibodies targeting IL8 and TNF [16–18], they should be evaluated as predictive enrichment factors for their designated therapies in future trials.

## Conclusions

In summary, we found that plasma levels of sTNFR1 and IL8 consistently identified sepsis patients at higher risk of mortality and might be useful as prognostic enrichment factors in future trials by improving trial efficiency and power and reducing the number of survivors unnecessarily exposed to potentially risky therapy.

## Supplementary information

**Additional file 1.** Supplemental Methods.

**Additional file 2.** Supplemental Data.

## Data Availability

The datasets analyzed during the current study are available from the corresponding author upon reasonable request.

## Abbreviations

sTNFR1: Soluble tumor necrosis factor receptor 1
IL8: Interleukin 8
Ang2: Angiopoietin-2
RD: Risk difference
PENN: University of Pennsylvania
UCSF: University of California San Francisco
APACHE: Acute Physiology, Age, Chronic Health Evaluation
ARDS: Acute respiratory distress syndrome
ICU: Intensive care unit
AUC: Area under the receiver operating characteristic curve
CI: Confidence interval
DCA: Decision curve analysis
OR: Odds ratio
PPV: Positive predictive value
NPV: Negative predictive value
