# Supplementary material for: Plasma sTNFR1 and IL8 for prognostic enrichment in sepsis trials: a prospective cohort study
Source: Crit Care. 2019 Dec 9;23:400. doi: 10.1186/s13054-019-2684-2 (PMC6902425; doi:10.1186/s13054-019-2684-2)
Supplement: Supplementary file 1 — Additional file 1. Supplemental Methods. [file 13054_2019_2684_MOESM1_ESM.pdf]

Supplemental Methods

Plasma sTNFR1 and IL8 for Prognostic Enrichment in Sepsis Trials: A Prospective Cohort Study

Anderson BJ, Calfee CS, Liu KD, Reilly JP, Kangelaris KN, Shashaty MGS, Lazaar AL, Bayliffe AI, Miano TA, Gallop RJ, Dunn TG, Johansson E, Abbott J, Jauregui A, Deiss T, Vessel K, Belzer A, Zhou H, Matthay MA, Meyer NJ, and Christie JD.

Study design

We performed a multicenter prospective cohort study enrolling critically ill patients with sepsis admitted from the emergency department (ED) at two academic university hospitals, the University of Pennsylvania and the University of California San Francisco. We chose *a priori* to enroll 200 subjects at each site during the same enrollment period to improve harmonization. Patients were followed for 30 days for mortality. Each cohort was approved by the Institutional Review Board (IRB) at their respective institution.

University of Pennsylvania (PENN) cohort

Patients were enrolled in the Molecular Epidemiology of SepsiS in the ICU (MESSI) cohort between September 2012 – December 2015. Inclusion criteria were admission to the intensive care unit (ICU) from the emergency department (ED) with a strongly suspected infection and new organ failure, consistent with Sepsis-3 definitions (1). Exclusion criteria included a non-infectious reason for ICU admission or sepsis not being the primary reason for ICU admission; do not attempt resuscitation, do not intubate, or comfort measures only preference at the time of ICU admission; residence in a long-term acute care hospital; or prior enrollment in the MESSI study during a prior ICU admission. Confirmation of a diagnosis of sepsis and the source of sepsis was determined through review by study investigators. Subjects were enrolled with a waiver of timely informed consent with the approval of the University of Pennsylvania Institutional Review Board (IRB). All patients or their surrogates provided informed consent with the exception of patients who expired before they or their surrogate could be approached for consent.

University of California, San Francisco (UCSF) cohort

Patients eligible for this analysis were enrolled in the Early Assessment of Renal and Lung Injury (EARLI) cohort between September 2012 – October 2015. Inclusion criteria for EARLI were admission to the ICU or the acute care ward. Exclusion criteria included an uncomplicated admission (i.e. anticipated ICU stay <24 hours, overdose or alcohol withdrawal, uncomplicated gastrointestinal bleed, diabetic ketoacidosis, admission for monitoring only); ICU admission >24hrs before screened (i.e. weekends and holidays); limitations on therapy (i.e. preference for comfort measures only at the time of admission, refusing care, declining blood draws); admission for trauma or an isolated neurologic or neurosurgical issue; or vulnerable populations (children, pregnancy women, admission for suicidality). For this analysis we included only patients enrolled in EARLI who were admitted to the ICU with a strongly suspected infection and at least two systemic inflammatory response syndrome (SIRS) criteria, consistent with Sepsis-2 criteria (2), and a final diagnosis of sepsis after review by study investigators. All patients or their surrogates provided informed consent, with the exception of patients who expired before they or their surrogate could be approached for informed consent, and patients whose critical illness precluded informed consent and for whom a surrogate could not be identified after 28 days; for these patients, a waiver of informed consent was approved by the IRB of the University of California, San Francisco.

Data collection and definitions

Clinical variables were abstracted from the medical record by trained study personnel at each site. Physician investigators at each site confirmed sepsis criteria and adjudicated site of infection. Acute Physiology, Age and Chronic Health Evaluation (APACHE) II scores were calculated based on data within the first 24 hours of ICU admission (3). Acute respiratory distress syndrome was defined within 6 days of admission using the Berlin definition (4), with chest radiographs interpreted by physician investigators at each site. Immunocompromised status was defined by APACHE criteria (3, 5). Specifically, patients were defined as immunocompromised if they had a diagnosis of leukemia, lymphoma, metastatic malignancy, multiple myeloma, human

acquired immunodeficiency syndrome or if they received immunosuppressive therapy, high-dose corticosteroids, chemotherapy, or radiation. We defined septic shock as the receipt of vasopressors and lactate level > 2.0 mmol/L on the day of intensive care unit (ICU) admission, consistent with Sepsis-3 consensus criteria (1).

Plasma protein measurement

Plasma samples were obtained as close to the time of ICU bed request as feasible and within 24 hours of ICU admission. Samples were frozen at -80°C until analysis. The concentrations of soluble tumor necrosis factor receptor-1 (sTNFR1), interleukin-8 (IL8) and Angiopoietin-2 (Ang2) in ICU admission plasma were measured in duplicate by lab personnel blinded to outcome assessments using commercially available enzyme-linked immunosorbent assays (R&D Systems, Minneapolis MN). The lower limit of detection (7.5pg/ml) was used for patients who had undetectable IL8 plasma concentrations at ICU admission (n=11).

Statistical Methods

Baseline comparisons were made using χ^2 or rank-sum tests as appropriate. We assessed for nonlinear exposure-outcome relationships with inspection of locally weighted scatterplot smoothing curves and log-transformed biomarker concentrations prior to model inclusion (6). We first confirmed that each marker had prognostic value by determining whether the marker was independently associated with mortality when adjusted for clinical variables, and by determining whether the marker improved model fit (likelihood ratio test) and discrimination (area under the receiver operating characteristic curve [AUC] using the DeLong method (7)) when added to a clinical variable model for mortality. We chose clinical variables that were easily available at the time of admission and associated with mortality, including age, diabetes, cirrhosis, immunocompromised status, septic shock at presentation, and invasive mechanical ventilation at presentation. Next, we sought to operationalize each marker for simple use as potential enrichment factors. We split the population according to the site of enrollment and derived thresholds for each marker in the derivation population (University of Pennsylvania) using the Youden method (8). We performed multivariable

logistic regression and regression risk analysis to calculate standardized risks and risk differences for 30-day mortality between marker-positive and marker-negative subjects (9, 10). In the derivation population we adjusted for the clinical variables detailed above. We then validated each marker threshold in the validation population (University of California San Francisco). We simulated the effect of simply applying the marker threshold in an external population and focused on whether the unadjusted risk difference in the validation population fell within the 95% confidence interval of the standardized risk difference observed in the derivation population, indicating the marker identified a high-risk subgroup in a similar fashion in each population while accounting for differences in baseline mortality (11). We secondarily performed multivariable analysis in the validation population to ensure our results were robust to adjustment, and tested whether each dichotomous marker improved model fit and discrimination in the individual cohorts when added to a clinical variable model for 30-day mortality using the methods detailed above. We also tested for effect modification by immunocompromised status in all analyses because differences in circulating immunomodulatory molecules have been reported in these patients (12).

Next, we combined the two populations after confirming there was no evidence of effect modification by site and employed decision curve analysis (DCA) to illustrate the net benefit of each biomarker enrichment strategy and simulate the effect of the strategy on sample size requirements for a theoretical clinical trial (13-15). To perform DCA, the net benefit of each enrollment strategy was calculated at specific threshold probabilities using the formula (true positives/n) - (false positive/n) * (p_t/1-p_t), where the threshold probability p_t represents the mortality risk that a clinical trial would seek as criteria for enrollment. For example, a trial might seek to enroll patients with at least a 35% risk of mortality. The net benefit varies with the threshold probability, since the threshold probability incorporates the harm of missing non-survivors (false negatives) and enrolling too many survivors (false positives). We focused on threshold probabilities between 15-50%, because enrichment is unnecessary at low thresholds given baseline sepsis mortality rates, and we reasoned that patients with >50% mortality risk may be excluded from trials because they may be less likely to respond to therapy. The decision curves are interpreted vertically, such that at each mortality risk threshold, the strategy

with the highest net benefit is the strategy that selects the highest number of true positives and therefore is the optimal strategy to identify a high-risk subgroup for trial enrollment. To provide tangible examples of the benefits of prognostic enrichment, we also calculated sample sizes needed for a hypothetical trial with 90% power testing a therapy with a 20% relative risk reduction of mortality with type I error of 5%.

First, we used DCA to compare enrichment strategies based on individual biomarkers. Specifically, we compared 1) enrolling all patients with sepsis, 2) enrolling only patients with septic shock, or 3) enrolling only patients positive for a single marker. We subsequently used DCA to compare enrichment strategies based on multiple biomarkers. Specifically, we compared enrolling only patients positive for more than one marker and enrolling only patients whose predicted mortality was above a certain threshold in a two biomarker model treating the biomarkers as continuous variables. We tested different cutoffs for the predicted mortality in the two biomarker continuous model and a predicted mortality of >35% performed best and was included in the DCA. We chose septic shock as our primary clinical variable enrichment method because it is often used to identify a high-risk subgroup in sepsis trials (16,17). Secondarily, we evaluated an APACHE II score ≥20, indicating a predicted mortality ≥ 40% (18), and a peak lactate ≥4 mmol/L within the first 24 hours, indicating a predicted mortality ≥35% (19), as potential prognostic enrichment factors.

Analyses were performed using Stata version 15.1 (College Station, TX). A two-sided $p < 0.05$ was considered statistically significant.

References cited in online supplement:

1. Singer M, Deutschman CS, Seymour CW, Shankar-Hari M, Annane D, Bauer M. The Third International Consensus Definitions for Sepsis and Septic Shock (Sepsis-3). *JAMA* 2016; 315.
2. Bone RC, Balk RA, Cerra FB, Dellinger RP, Fein AM, Knaus WA, Schein RM, Sibbald WJ. Definitions for sepsis and organ failure and guidelines for the use of innovative therapies in sepsis. The ACCP/SCCM Consensus Conference Committee. American College of Chest Physicians/Society of Critical Care Medicine. *Chest* 1992; 101: 1644-1655.
3. Knaus WA, Wagner DP, Draper EA, Zimmerman JE, Bergner M, Bastos PG, Sirio CA, Murphy DJ, Lotring T, Damiano A, et al. The APACHE III prognostic system. Risk prediction of hospital mortality for critically ill hospitalized adults. *Chest* 1991; 100: 1619-1636.
4. Force ADT, Ranieri VM, Rubenfeld GD, Thompson BT, Ferguson ND, Caldwell E, Fan E, Camporota L, Slutsky AS. Acute respiratory distress syndrome: the Berlin Definition. *JAMA* 2012; 307: 2526-2533.
5. Knaus WA, Draper EA, Wagner DP, Zimmerman JE. APACHE II: a severity of disease classification system. *Crit Care Med* 1985; 13: 818-829.
6. Cleveland WS. Robust Locally Weighted Regression and Smoothing Scatterplots. *J Am Stat Assoc* 1979; 74: 829-836.
7. DeLong ER, DeLong DM, Clarke-Pearson DL. Comparing the areas under two or more correlated receiver operating characteristic curves: a nonparametric approach. Biometrics 1988;44(3):837-845.
8. Youden WJ. Index for rating diagnostic tests. *Cancer* 1950; 3: 32-35.
9. Kleinman LC, Norton EC. What's the Risk? A simple approach for estimating adjusted risk measures from nonlinear models including logistic regression. *Health Serv Res* 2009; 44: 288-302.

11\. Norton EC, Miller MM, Kleinman LC. Computing adjusted risk ratios and risk differences in Stata. *Stata J* 2013; 13: 492-509.

11\. Ryan CM, Schoenfeld DA, Thorpe WP, Sheridan RL, Cassem EH, Tompkins RG. Objective estimates of the probability of death from burn injuries. *N Engl J Med* 1998; 338: 362-366.

12\. Reilly JP, Anderson BJ, Hudock KM, Dunn TG, Kazi A, Tommasini A, Charles D, Shashaty MG, Mikkelsen ME, Christie JD, Meyer NJ. Neutropenic sepsis is associated with distinct

clinical and biological characteristics: a cohort study of severe sepsis. *Crit Care* 2016; 20: 222.

13. Vickers AJ. Decision analysis for the evaluation of diagnostic tests, prediction models and molecular markers. *Am Stat* 2008; 62: 314-320.
14. Vickers AJ, Cronin AM. Traditional statistical methods for evaluating prediction models are uninformative as to clinical value: towards a decision analytic framework. *Semin Oncol* 2010; 37: 31-38.
15. Vickers AJ, Van Calster B, Steyerberg EW. Net benefit approaches to the evaluation of prediction models, molecular markers, and diagnostic tests. *BMJ* 2016; 352: i6.
16. Russell JA, Walley KR, Singer J, Gordon AC, Hebert PC, Cooper DJ, Holmes CL, Mehta S, Granton JT, Storms MM, Cook DJ, Presneill JJ, Ayers D, Investigators V. Vasopressin versus norepinephrine infusion in patients with septic shock. *N Engl J Med* 2008; 358: 877-887.
17. Caironi P, Tognoni G, Masson S, Fumagalli R, Pesenti A, Romero M, Fanizza C, Caspani L, Faenza S, Grasselli G, Iapichino G, Antonelli M, Parrini V, Fiore G, Latini R, Gattinoni L, Investigators AS. Albumin replacement in patients with severe sepsis or septic shock. *N Engl J Med* 2014; 370: 1412-1421.
18. Knaus WA, Draper EA, Wagner DP, et al. APACHE II: a severity of disease classification system. Crit Care Med 1985;13(10):818-829.
19. Shankar-Hari M, Phillips GS, Levy ML, et al. Developing a New Definition and Assessing New Clinical Criteria for Septic Shock: For the Third International Consensus Definitions for Sepsis and Septic Shock (Sepsis-3). JAMA 2016;315(8):775-787.
