# Supplementary material for: Plasma sTNFR1 and IL8 for prognostic enrichment in sepsis trials: a prospective cohort study
Source: Crit Care. 2019 Dec 9;23:400. doi: 10.1186/s13054-019-2684-2 (PMC6902425; doi:10.1186/s13054-019-2684-2)

Supplemental Data

Plasma sTNFR1 and IL8 for Prognostic Enrichment in Sepsis Trials: A Prospective Cohort Study

Anderson BJ, Calfee CS, Liu KD, Reilly JP, Kangelaris KN, Shashaty MGS, Lazaar AL, Bayliffe AI, Miano TA, Gallop RJ, Dunn TG, Johansson E, Abbott J, Jauregui A, Deiss T, Vessel K, Belzer A, Zhou H, Matthay MA, Meyer NJ, and Christie JD.

Supplemental Figure S1. Consort diagram of patient enrollment and 30-day mortality at the University of Pennsylvania and University of California San Francisco.

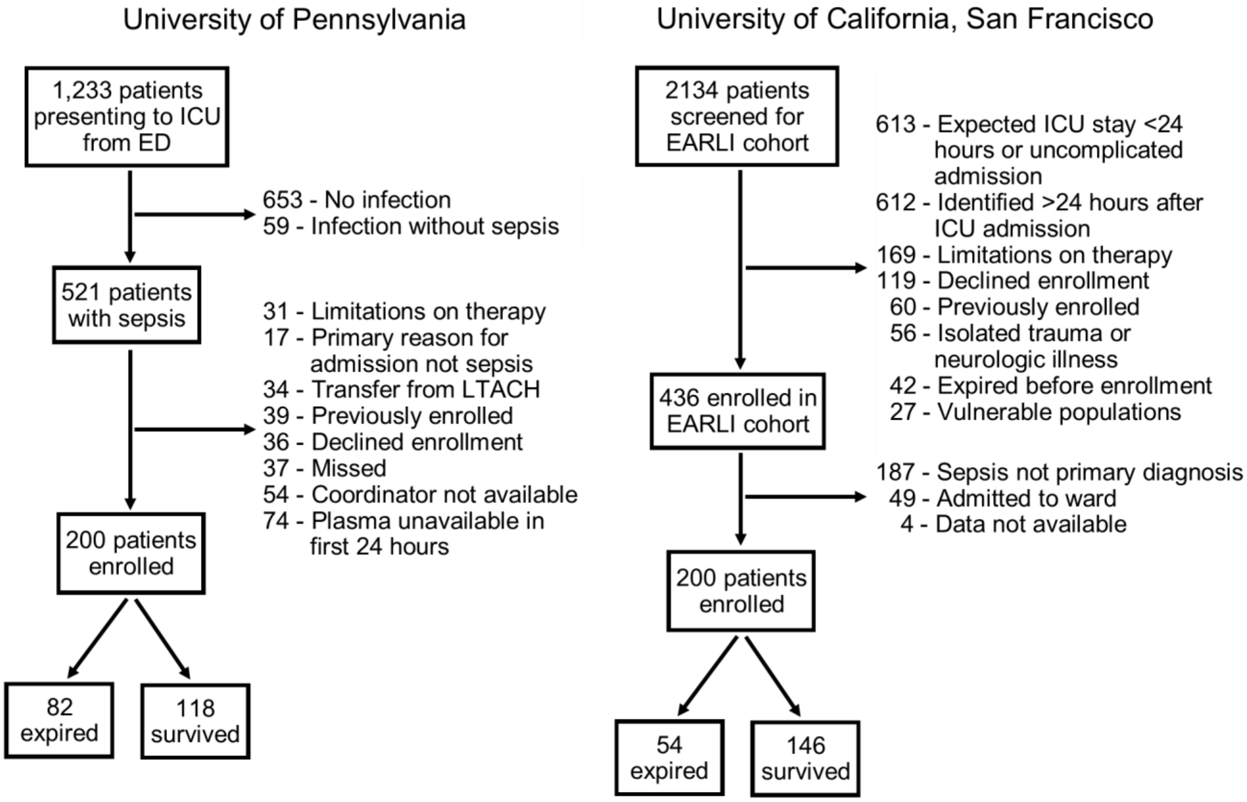

Supplemental Table S1. Confirmation of the prognostic relevance of each marker was assessed by determining the independent association of each marker with 30-day mortality when adjusting for age, diabetes, cirrhosis, immunocompromised status, septic shock at presentation and invasive mechanical ventilation at presentation. Model fit was assessed using the likelihood ratio test and discrimination was assessed using the area under the receiver operating characteristic curve (AUC), when each marker was added to a clinical variable model for 30-day mortality that included age, diabetes, cirrhosis, immunocompromised status, septic shock at presentation and invasive mechanical ventilation at presentation.

	**Association with mortality**	**Model Fit**		**Discrimination**		
**Marker**	**OR per 1-log increase**	χ^2	**p value**	**Clinical variables alone**	**Clinical variables with marker**	**p value**
sTNFR1	1.68 (1.23-2.28)	11.5	<0.001	0.701 (0.649-0.754)	0.724 (0.672-0.775)	0.099
IL8	1.25 (1.09-1.43)	11.0	<0.001	0.701 (0.649-0.754)	0.720 (0.669-0.770)	0.15
Ang2	1.53 (1.16-2.01)	9.9	0.002	0.701 (0.649-0.754)	0.718 (0.667-0.769)	0.20

Supplemental Table S2. Comparison of model fit using the likelihood ratio (LR) test and discrimination using the area under the receiver operating characteristic curve (AUC) when adding each marker threshold to a clinical variable model for 30-day mortality that includes age, diabetes, cirrhosis, immunocompromised status, septic shock at presentation and invasive mechanical ventilation at presentation. The IL-8 analysis is limited to immunocompetent patients only.

		**Goodness of Fit (LR test)**		**AUC Comparison**		
**Marker**	**Population**	χ^2	**p value**	**Clinical variables alone**	**Clinical variables with marker**	**p value**
**sTNFR1**	Derivation	9.73	0.002	0.708	0.740	0.15
	Validation	4.23	0.040	0.748	0.757	0.54
**IL-8**	Derivation	4.35	0.037	0.773	0.793	0.45
	Validation	9.06	0.003	0.728	0.769	0.15
**Ang-2**	Derivation	8.07	0.005	0.708	0.739	0.12
	Validation	2.98	0.085	0.748	0.755	0.57

Supplemental Table S3. Characteristics of enrichment factors in the combined population (N=400).

Variable(s)	Number (%) of subjects with enrichment factor	Positive predictive value (95% CI)	False positive rate (95% CI)	Negative predictive value (95% CI)	False negative rate (95% CI)	Risk difference (95% CI) of 30-day mortality when factor present
Septic shock at presentation	175 (43.8%)	42.3% (34.9, 50.0)	57.7% (50.0, 65.1)	72.4% (66.1, 33.9)	27.6% (21.8, 33.9)	14.7% (5.4, 24.1)
sTNFR1 positive	160 (40.0%)	48.8% (40.8, 56.8)	51.2% (43.2, 59.2)	75.8% (70.0, 81.1)	24.2% (18.9, 30.1)	24.6% (15.1, 34.0)
Ang-2 positive	266 (66.5%)	40.6% (34.6, 46.8)	59.4% (52.3, 65.4)	79.1% (71.2, 85.6)	20.9% (14.4, 28.8)	19.7% (10.6, 28.8)
APACHE II Score >20	298 (74.5%)	41.3% (35.6, 47.1)	58.7% (52.9, 64.4)	87.2% (79.2, 93.0)	12.8% (6.9, 20.8)	28.5% (20.0, 37.1)
Lactate >4 mmol/L within first 24 hours of admission	144 (36.8%)	45.8% (37.5, 54.3)	54.2% (45.7, 62.5)	72.9% (66.9, 78.3)	27.1% (21.7, 33.1)	18.7% (8.9, 28.6)

Supplemental Table S4. Characteristics of enrichment factors in the combined population restricted to immunocompetent subjects (N=278).

Variable(s)	Number (%) of subjects with enrichment factor	Positive predictive value (95% CI)	False positive rate (95% CI)	Negative predictive value (95% CI)	False negative rate (95% CI)	Risk difference (95% CI) of 30-day mortality when factor present
Septic shock at presentation	125 (45.0%)	38.4% (29.8, 47.5)	61.6% (52.5, 70.2)	77.1% (69.6, 83.5)	22.9% (21.8, 33.9)	15.5% (4.7, 26.3)
IL-8 positive	125 (45.0%)	43.2% (34.4, 52.4)	56.8% (47.6, 65.6)	81.0% (73.9, 86.9)	19.0% (13.1, 26.1)	24.3% (13.6, 34.9)

Supplemental Table S5. Net benefit and reduction in the number of survivors unnecessarily exposed to a clinical trial when enrolling only sTNFR1 positive patients or enrolling only patients with septic shock across a range of mortality risk thresholds. The shaded strategy is the optimal enrichment strategy at the specific risk threshold.

		Threshold probability for sepsis mortality							
**Analytic method**	**Enrichment method**	15%	20%	25%	30%	35%	40%	45%	50%
Net benefit	All sepsis	0.223	0.175	0.120	0.057	-0.015	-0.100	-0.200	-0.320
	Septic shock	0.140	0.122	0.101	0.077	0.049	0.017	-0.022	-0.068
	sTNFR1	0.159	0.144	0.127	0.107	0.085	0.058	0.027	-0.010
Reduction in survivors exposed per 100 patients	All sepsis	Reference							
	Septic shock	---	---	---	4.6	12.0	17.5	21.8	25.3
	sTNFR1	---	---	2.0	11.7	18.6	23.8	27.8	31.0

Supplemental Table S6. Unadjusted risk differences of 30-day mortality categorized by IL-8 positivity, and categorized by site and immunocompromised status.

	Number (%) of subjects above threshold	30-day mortality if below threshold	30-day mortality if above threshold	Risk difference of 30-day mortality if above threshold	p
All patients					
Derivation (n=200)	115 (57.5%)	30.3% (21.6, 39.0)	52.0% (42.5, 61.6)	21.7% (8.5, 35.0)	0.001
Validation (n=200)	78 (39.0%)	21.1% (14.1, 28.0)	38.8% (27.1, 50.5)	17.8% (4.2, 31.3)	0.010
Immunocompetent					
Derivation (n=105)	57 (54.3%)	22.9% (11.0, 34.8)	42.1% (29.3, 54.9)	19.2% (1.7, 36.7)	0.032
Validation (n=173)	68 (39.3%)	17.1% (9.9, 24.4)	44.1% (32.3, 55.9)	27.0% (13.2, 40.8)	<0.001
Immunocompromised					
Derivation (n=95)	58 (61.1%)	46.0% (29.9, 62.0)	51.7% (38.9, 64.6)	5.8% (-14.8, 26.4)	0.58
Validation (n=27)	10 (37.0%)	29.4% (7.8, 51.1)	10.0% (-8.6, 28.6)	-19.4% (-48.0, 9.1)	0.18

Supplemental Table S7. Net benefit and reduction in the number of survivors unnecessarily exposed to a clinical trial when enrolling only IL-8 positive patients or enrolling only patients with septic shock across a range of mortality risk thresholds. The shaded strategy is the optimal enrichment strategy at the specific risk threshold. Analysis is limited to immunocompetent patients only.

		Threshold probability for sepsis mortality							
**Analytic method**	**Enrichment method**	15%	20%	25%	30%	35%	40%	45%	50%
Net benefit	All sepsis	0.175	0.123	0.065	-0.002	-0.079	-0.169	-0.275	-0.403
	Septic shock	0.123	0.103	0.080	0.054	0.024	-0.012	-0.054	-0.104
	IL-8	0.149	0.130	0.109	0.085	0.057	0.024	-0.015	-0.061
Reduction in survivors exposed per 100 patients	All sepsis	Reference							
	Septic shock	---	---	4.7	13.1	19.1	23.6	27.1	29.9
	IL-8	---	2.9	13.3	20.3	25.2	29.0	31.9	34.2

Supplemental Table S8. Net benefit and reduction in the number of survivors unnecessarily exposed to a clinical trial when enrolling only Ang-2 positive patients or enrolling only patients with septic shock across a range of mortality risk thresholds. The shaded strategy is the optimal enrichment strategy at the specific risk threshold.

		Threshold probability for sepsis mortality							
**Analytic method**	**Enrichment method**	15%	20%	25%	30%	35%	40%	45%	50%
Net benefit	All sepsis	0.223	0.175	0.120	0.057	-0.015	-0.100	-0.200	-0.320
	Septic shock	0.140	0.122	0.101	0.077	0.049	0.017	-0.022	-0.068
	Ang2	0.200	0.171	0.138	0.101	0.057	0.007	-0.053	-0.125
Reduction in survivors exposed per 100 patients	All sepsis	Reference							
	Septic shock	---	---	---	4.6	12.0	17.5	21.8	25.3
	Ang2	---	---	5.5	10.2	13.5	16.0	17.9	19.5

Supplemental Table S9. Crude risk differences for 30-day mortality and discrimination for 30-day mortality using the area under the receiver operating characteristic curve (AUC) when adding each biomarker variable to a clinical variable model for 30-day mortality that includes age, diabetes, cirrhosis, immunocompromised status, septic shock at presentation and invasive mechanical ventilation at presentation in the combined cohort (N=400).

**Variable(s)**	**Risk difference of 30-day mortality**	**AUC when added to a of clinical variable model for mortality**
sTNFR1 positive	24.6% (15.1, 34.0)	0.727 (0.676, 0.779)
IL-8 positive	19.4% (10.3, 28.5)	0.732 (0.682, 0.782)
Ang-2 positive	19.7% (10.6, 28.8)	0.722 (0.670, 0.773)
Combined positivity for sTNFR1 and IL-8	26.7% (16.3, 37.2)	0.735 (0.685, 0.785)
Predicted mortality >30% from a model using sTNFR1 and IL-8 as continuous variables	20.6% (11.7, 29.6)	0.723 (0.672, 0.774)
Predicted mortality >35% from a model using sTNFR1 and IL-8 as continuous variables	23.0% (13.8, 32.2)	0.727 (0.676, 0.777)
Predicted mortality >40% from a model using sTNFR1 and IL-8 as continuous variables	22.5% (12.3, 32.8)	0.719 (0.668, 0.770)

Supplemental Figure S2. (A) Net benefit and (B) reduction in the number of survivors unnecessarily exposed to a clinical trial when enrolling only patients who are sTNFR1 positive, enrolling only patients who are IL-8 positive, and enrolling only patients that are positive for both sTNFR1 and IL-8.

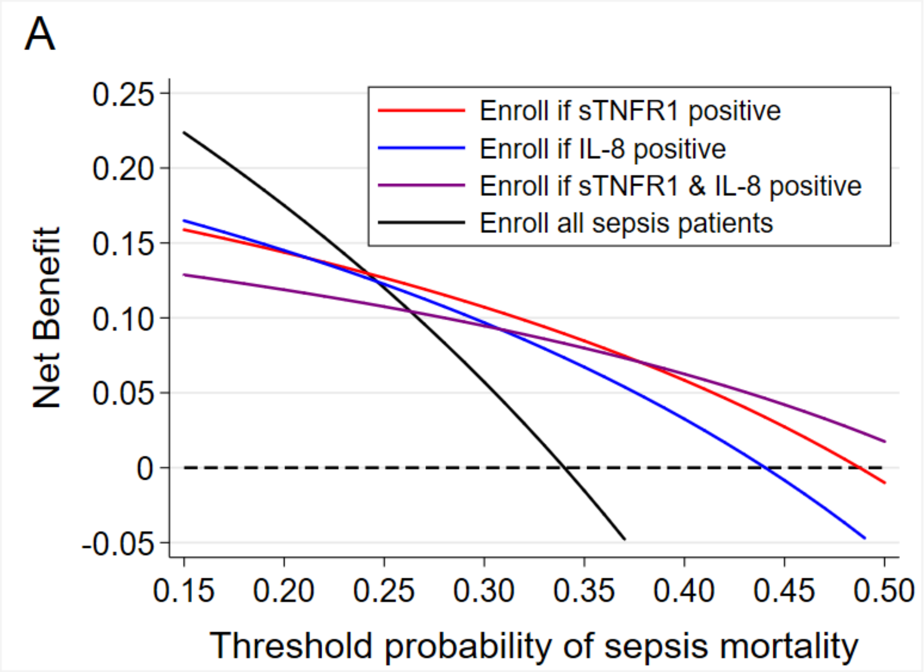

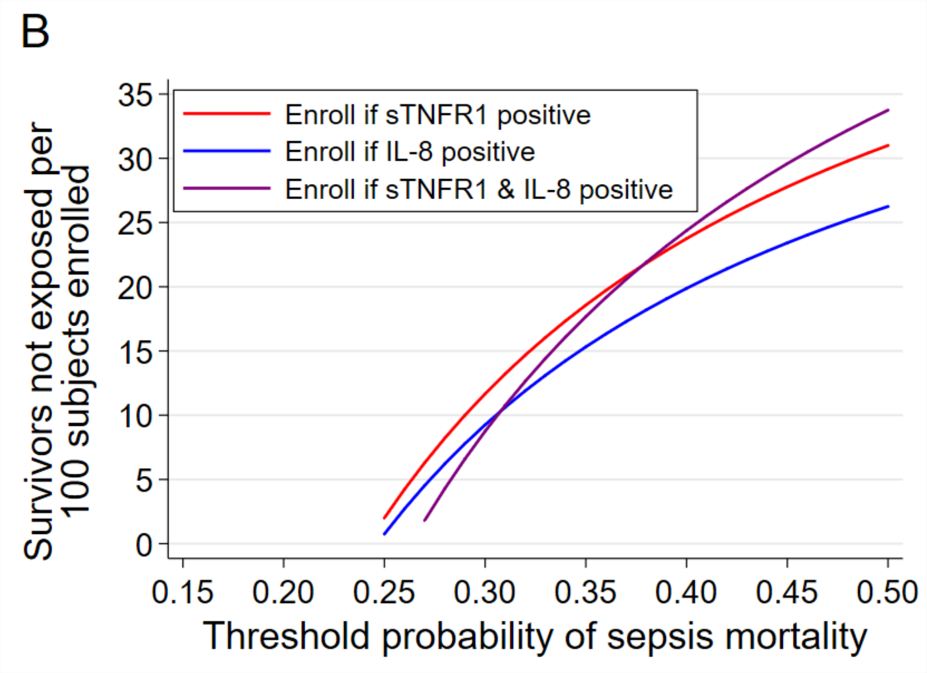

Supplemental Figure S3. (A) Net benefit and (B) reduction in the number of survivors unnecessarily exposed to a clinical trial when enrolling immunocompetent patients who are sTNFR1 positive, enrolling immunocompetent patients who are IL-8 positive, and enrolling immunocompetent patients positive for both sTNFR1 and IL-8.

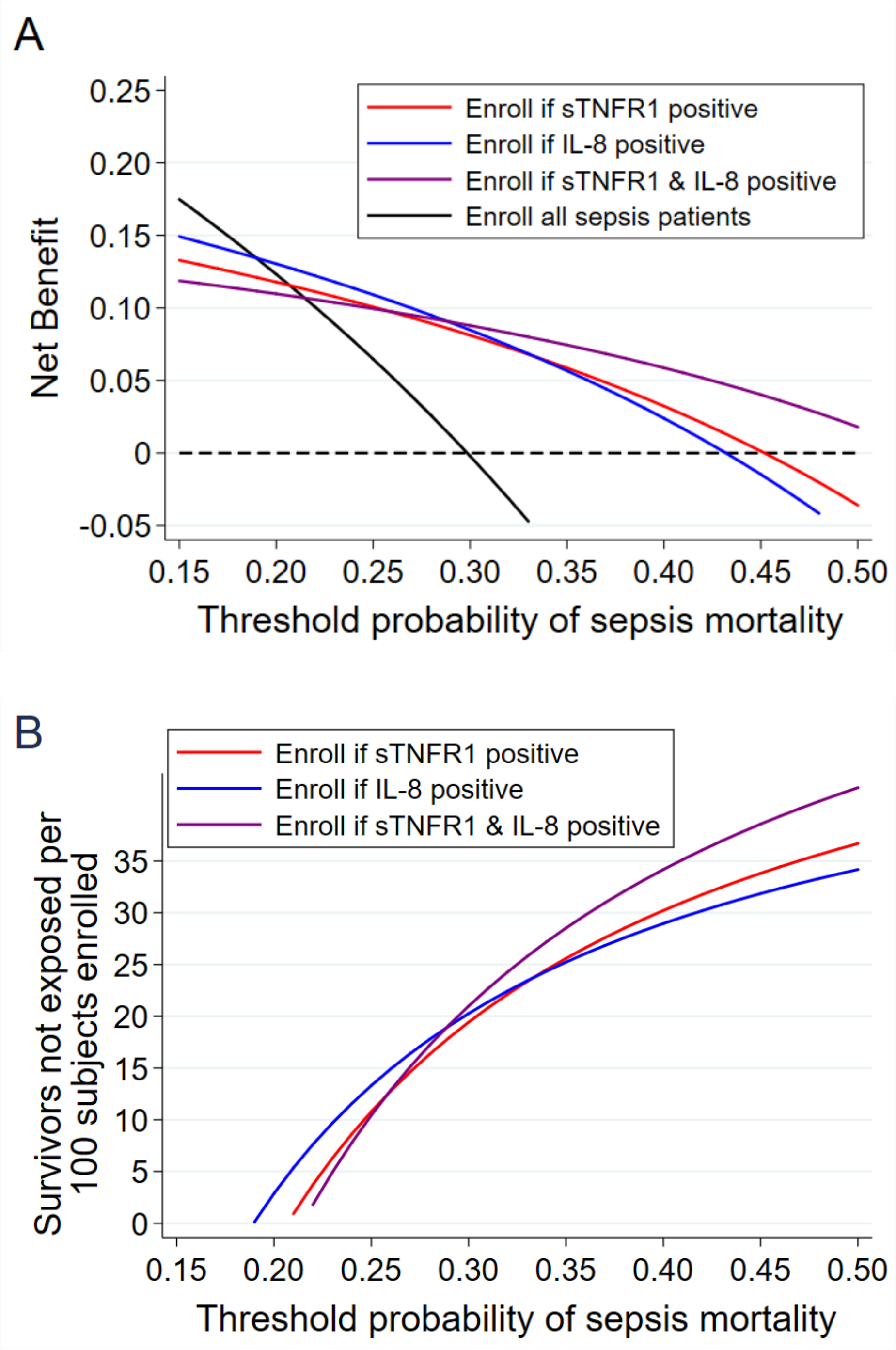

Supplemental Figure S4. (A) Net benefit and (B) reduction in the number of survivors unnecessarily exposed to a clinical trial when enrolling only patients who are sTNFR1 positive, enrolling only patients who are IL-8 positive, and enrolling only patients with a predicted mortality >35% based on a two biomarker model using sTNFR1 and IL-8 as continuous variables.

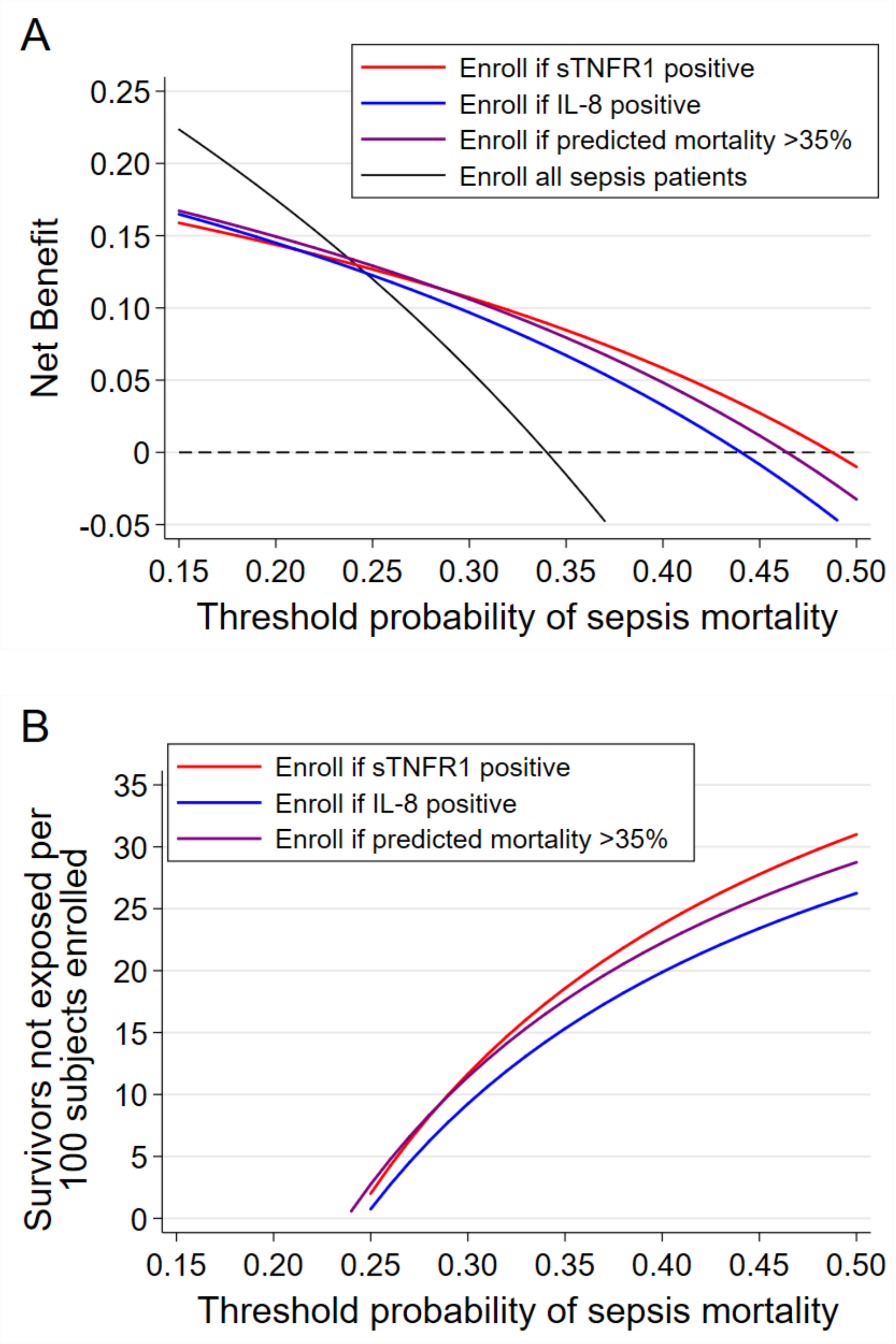

Supplemental Figure S5. (A) Net benefit and (B) reduction in the number of survivors unnecessarily exposed to a clinical trial when enrolling only patients who are sTNFR1 positive, enrolling only patients who are IL-8 positive, and enrolling only patients with an APACHE II score >20.

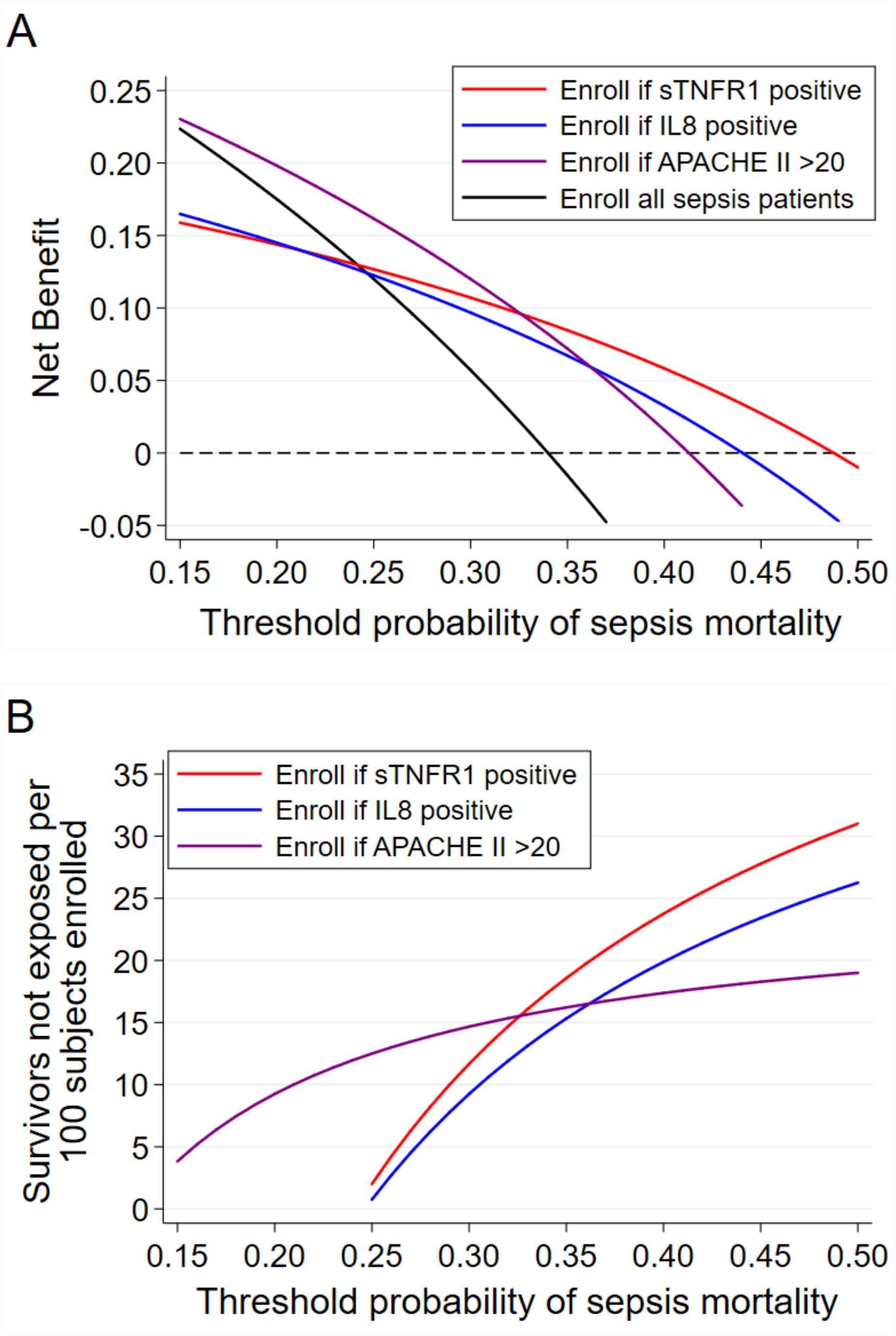

Supplemental Figure S6. (A) Net benefit and (B) reduction in the number of survivors unnecessarily exposed to a clinical trial when enrolling only patients who are sTNFR1 positive, enrolling only patients who are IL-8 positive, and enrolling only patients with a lactate >4 mmol/L within the first 24 hours of ICU admission.

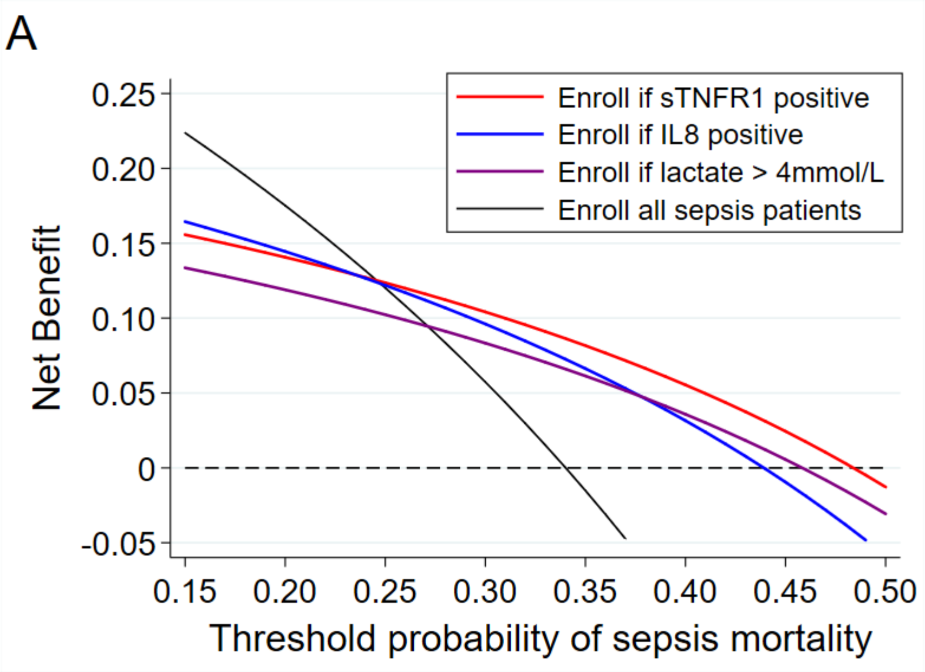

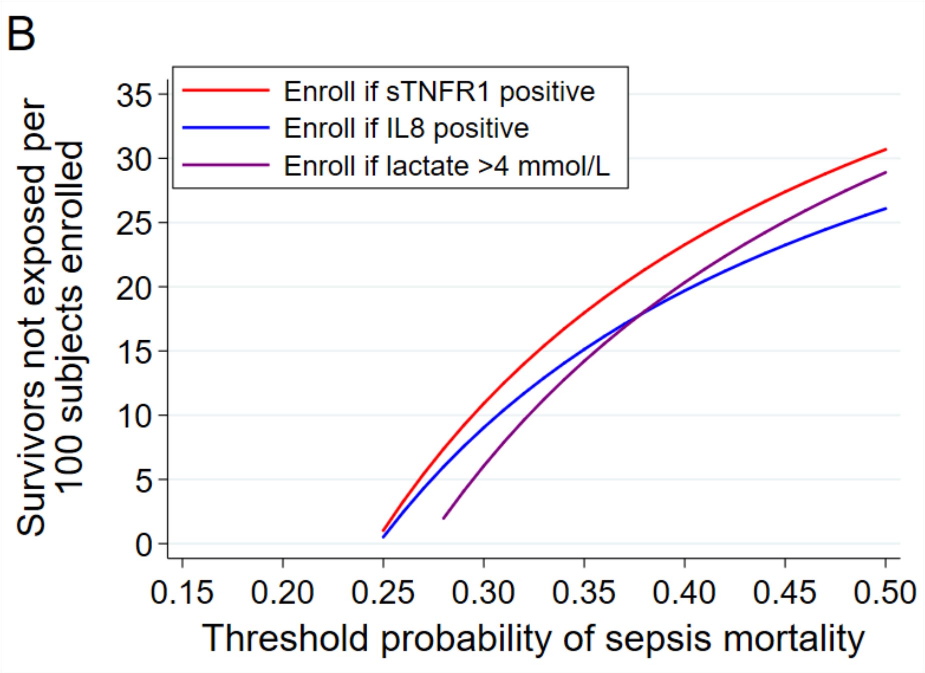

Supplement: Supplementary file 2 — Additional file 2. Supplemental Data. [file 13054_2019_2684_MOESM2_ESM.pdf]
